# Retro-Auricular Approach to the Fractures of the Mandibular Condyle: A Systematic Review

**DOI:** 10.3390/jcm10020230

**Published:** 2021-01-11

**Authors:** Maciej Sikora, Maciej Chęciński, Dariusz Chlubek

**Affiliations:** 1 Department of Maxillofacial Surgery, Hospital of the Ministry of Interior, Wojska Polskiego 51, 25-375 Kielce, Poland; sikora-maciej@wp.pl; 2 Department of Biochemistry and Medical Chemistry, Pomeranian Medical University, Powstańców Wielkopolskich 72, 70-111 Szczecin, Poland; 3 STOMADENT Non-Public Healthcare Institution, Dental Clinic, Kościuszki 32, 46-320 Praszka, Poland; maciej@checinscy.pl

**Keywords:** facial nerve injury, mandibular condyle fracture, mandibular head fracture, retro-auricular trans-meatal approach

## Abstract

This systematic review was conducted to evaluate the retro-auricular trans-meatal approach (RA) to mandibular head fractures. Fractures of the mandibular head (8%) are a specific type of mandibular condyle fractures (34%). Despite numerous complications of conservative treatment, e.g., limited mobility and even ankylosis of the temporomandibular joint, as well as shortening of the mandibular ramus resulting in malocclusion, surgical intervention in this type of fracture is still problematic. The main problems with the dominant pre-auricular approach are the high risk of paralysis of the facial nerve and persistence of a visible scar. An attractive alternative is RA, which, despite its long history, has been described in English very few times, i.e., in only two clinical trials described in three articles in the last 21 years. According to these studies, RA gives a minimum of 90% of ideal positions of bone fragments and an always fully preserved function of the facial nerve in the course of long-term observation. RA allows the application of long screws for fixation, which provide good stabilization. In addition, new types of headless screws leave a smooth, non-irritating bone surface, and the immediate future may be dominated by their resorbable varieties. RA can, therefore, be treated as a very favorable access to fractures of the mandibular head, especially due to the protection of the facial nerve and the possibility of providing a stable and predictable fixation.

## 1. Introduction

The mandible is a craniofacial bone in which the lower dental arch is embedded. It consists of the body and two rami. Each ramus ends with two processes: the coronoid process, which is the site of the attachment of a strong temporal muscle, and the condylar process in which the upper part is the articular head of the temporomandibular joint. Most fractures of the condylar process are indirect as a result of the force applied to the mandibular body. This type of injury is promoted by the mandible’s exposed position.

Despite the high incidence of condylar fractures and direct influence of such fractures on the temporomandibular joint functioning, the protocols for treatment of such fractures are still disputable. The most controversial is the treatment of the uppermost part of the condylar process of the mandible. These are specifically the entire mandibular head and the upper part of the mandibular neck.

**Figure 1:**
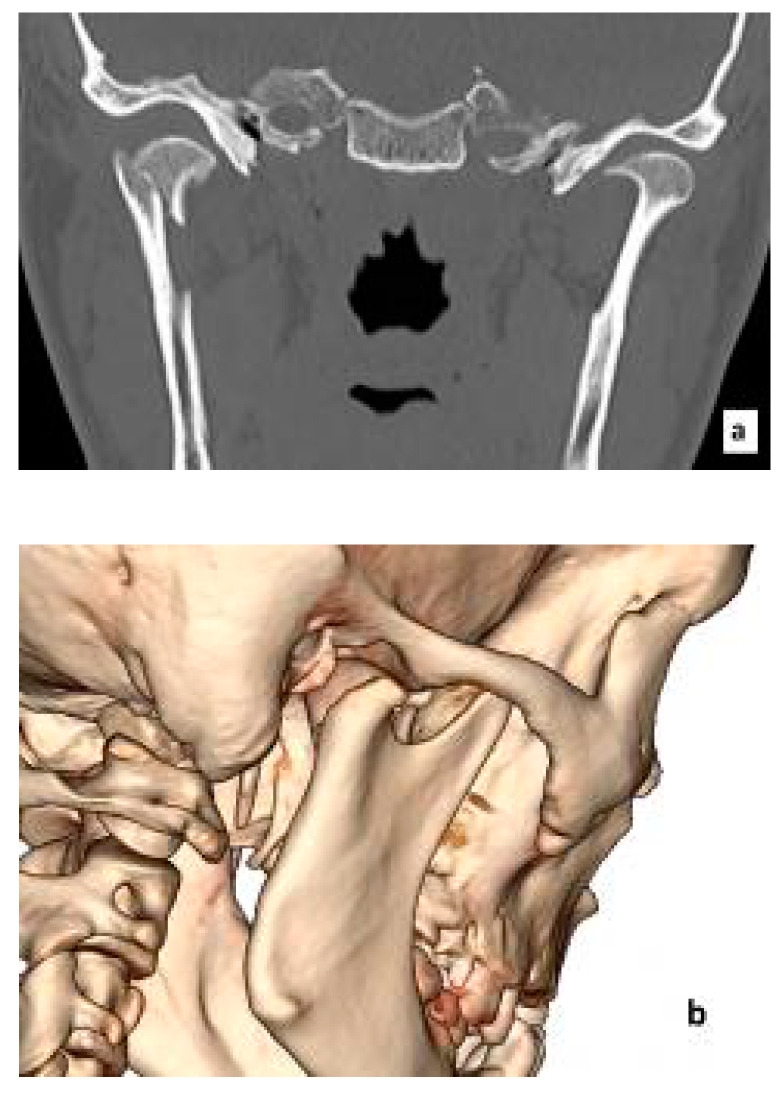
Fracture of the right mandibular head. Computed tomography: frontal scan (**a**) and 3D reconstruction (**b**).

**Figure 2:**
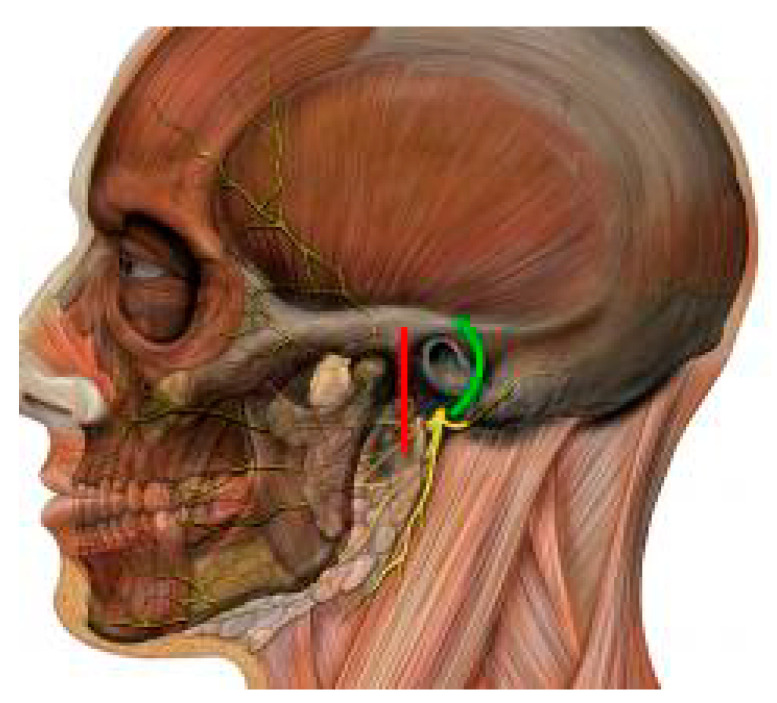
The course of the facial nerve branches in relation to the mandibular head. Retro-auricular (green line) and pre-auricular (red line) approaches. Modified from Patrick J. Lynch and C. Carl Jaffe: Head human skull lateral view, Head facial nerve superficial branches. 2006. https://creativecommons.org/licenses/by/2.5/.

Figure 1 shows an example of a mandibular head fracture in CT images. Access to these structures is prevented not only by the temporomandibular joint ligaments, but also by the very delicate facial nerve responsible for the facial muscles’ activity (Figure 2).

The following structures are located right beneath the preauricular area of the skin that is of great aesthetic importance. Gokkulakrishnan et al. note that the incidence of temporary facial nerve palsy resulting from iatrogenic injury during temporomandibular joint ankylosis procedures may be as high as 79% in the early post-operative period [1]. Such ankylosis is a potential complication of condylar head fractures regardless of whether they were treated (conservatively or surgically) or not. According to the data collected by Moin et al., the probability of an iatrogenic facial nerve injury depends on the procedure performed and, excluding ankylosis, in cases of temporomandibular joint surgery, it is up to 32% [2]. Such high values prompt to choose conservative methods or surgical procedures that allow this nerve to be bypassed.

While conservative treatment of condylar fractures is simpler, cheaper, and less invasive, it can cause numerous complications [3]. The previously mentioned complications may include dysfunctions of the temporomandibular joint, such as ankylosis, shortening of the mandibular rami resulting in facial deformation, deterioration of the gums and teeth due to the immobilization of the mandible, and significant limitations in carrying out hygienic procedures [4,5,6]. Conservative methods, however, have an undeniably bigger advantage over surgical ones by avoiding disruption of tissue continuity while creating access to the condyle. Therefore, the preferred treatment strategies so far have focused mainly on conservative management. Experts indicated that operating in these areas should be performed only by the most experienced surgeons [7].

However, over the course of a long-term follow-up, it is noted that the mandible’s mobility is lower in the patient group that has been treated conservatively in comparison with the patients that underwent the classic surgical procedure, giving 24%–40% lower interincisial opening as well as 19%–44% smaller lateral movements [8,9,10]. Moreover, the deviation of the mandible abducted after surgical treatment is proven to be negligible, and, in the conservative group, it may be statistically significant and up to one millimeter [9]. The pain determined by the Visual Analogue Scale (VAS) is lower for the surgical group [8,11,12]. In the study of Karan et al., the VAS-scale pain was approximately 1.0 after one week post-surgery compared to the conservative treatment group, in which patients at the same time rated it approximately at 3.5 [9]. The radiological evaluation confirms a higher percentage of perfect bone alignment with the use of surgical treatment [9,13,14]. In the study by Danda et al., the correct anatomical relationships were obtained in about a quarter of patients treated conservatively and in over 85% of patients who underwent surgery [14].

Comparing surgical and non-surgical methods of treating the mandibular head fractures is difficult and studies that reliably compare both strategies are still insufficient [8,9,10,11,12,13,14]. Despite that, some authors are trying to define the indications for each strategy [8,10]. The indications for surgical treatment of mandibular condyle fractures have changed over time. The 1983 Zide and Kent guidelines assumed the absolute necessity of surgical intervention in the presence of a foreign body, displacement of the condyle inside the cranial cavity or lateral extracapsular space, and the inability to obtain adequate occlusion [15]. These indications were maintained by adding more, which are treated, however, as recommendations, and not absolute ones, i.e., evident displacement of bone fragments, abnormal joint function, or the effects of trauma on the auditory structures [16]. As Chrcanovic points out, the indications for surgical management of diacapitular fractures are, in simplified terms, the loss of height of the mandibular branch and the displacement of the proximal part beyond the acetabulum [10]. The same author points out, however, that a significant number of mandibular head fractures can be treated conservatively. The indications for conservative treatment are defined as follows: (a) no shortening of the condyle height, (b) non-displaced fractures, (c) compound fractures with multiple fragments that are impossible to fixate, and (d) fractures in children [10]. As an example, in the work of Eckelt et al. concerning the results of treating various mandibular condylar fractures, the proportion of fractured mandibular heads treated conservatively and surgically was about half and half [9].

The study by Hirjak et al. described an analysis of the surgical treatment results for fractures of 28 mandibular heads from the preaural approach in 23 patients [11]. It allowed for assessing the position of the bone fragments one month after the operation. This position was 89% ideal or very close to anatomical due to the possibility of open reposition [11]. The improvement of anatomical relationships in 88% of cases of head fractures thanks to the use of open reduction was also noticed by McLeod et al. [12]. Difficulties with fixation of the mandibular comminuted heads were also emphasized by McLeod et al. [12] Moreover, these authors, in the group of 15 patients with 17 fractures of the mandibular heads operated by the preaural approach, observed three cases of facial nerve palsy with one of them being permanent [12]. In turn, Smolka et al. have not found a single case of persistent facial nerve paralysis in a group of 48 patients treated for a fracture of the mandibular head [13]. These authors also used a preauricular approach. Moreover, Smolka et al. noted the achievement of correct occlusion in all subjects. In a subgroup of 20 patients who were more closely observed, these authors determined a median mandibular abduction of 40 mm and a median laterotrusion of 7 mm on both sides [13]. The considerations on surgical access to mandibular head fractures in the context of alternative nonsurgical treatment are accurately summarized by Boffano et al. [8]. These authors emphasize that a golden standard in this field does not exist and surgical treatment in some centers is still considered experimental [8]. However, taking the risk of complications associated with surgery of the mandibular head into account, the clear benefits of correct reposition and stable fixation should not be forgotten [8].

Among surgical procedures, the retro-auricular approach (RA) bypasses the functional and aesthetic problems, i.e., facial nerve palsy and visible scar, thus, opening up a safer access to the uppermost mandibular condyle fractures. The RA used to manage condylar head fractures was first described exactly 100 years ago [17]. Nevertheless, about 20 years ago, the open reposition of such fractures was still considered an experimental method [7]. Over the last 20 years, many world authorities in the field of treatment of mandibular condylar fractures have confirmed the validity of the reposition and stabilization of the head fractures, recommending the RA [18,19,20]. This paper aims to present views from the years 2000–2020 on the use of RA in treating mandibular head fractures based on a systematic review of the literature.

## 2. Methods and Materials

PubMed and BASE (Bielefeld Academic Search Engine) engines were used to search medical databases. Searches based on keywords related to the retro-auricular approach were made on 9 November 2020. Further search for articles that are not indexed in the most popular medical databases has been carried out in two ways. In the first stage, the Google search engine implemented in the Google Scholar database has been used. Articles published before 2000 or in languages other than English were then rejected. Then, the references of all the remaining items were analyzed, which, taking the limitations above into account, did not give any further results. All stages of the systematic review were carried out in accordance with the PRISMA protocol (Preferred Reporting Items for Systematic Reviews and Meta-Analyzes) [21]. Cohen’s method was applied at each stage of the qualification.

Search keywords were selected based on the PICOS protocol (Population, Intervention, Comparison, Outcomes, Study Design), as shown in Table 1 [22]. Search strategies are detailed in Table S1. Medical databases and gray literature were reviewed by three researchers and authors of this article.

## 3. Results

**Figure 3:**
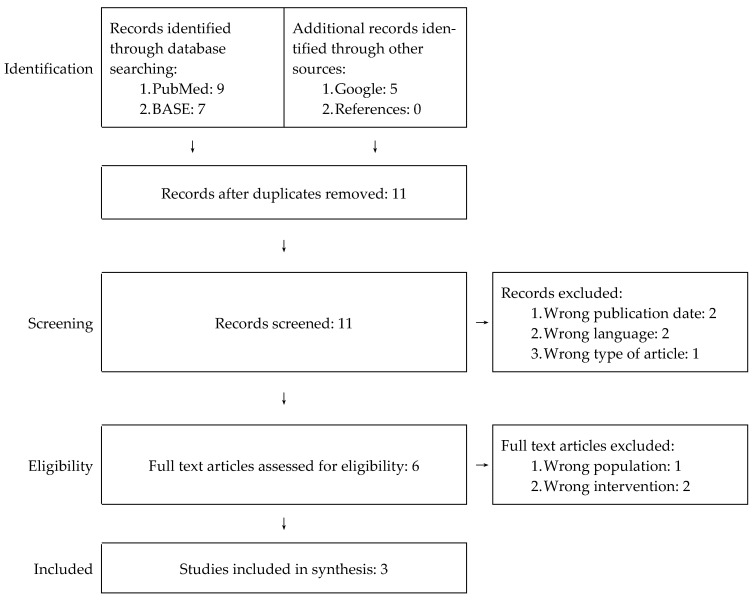
PRISMA flowchart for our systematic review.

A total of 21 items were identified of which 11 remained after eliminating duplicates. Out of 11 papers, eight were rejected during the screening and full-text reading stages, which did not meet the above-mentioned PICOS criteria. All authors of this review agreed independently on the decision to reject articles at the previously mentioned stages. The individual stages of the PRISMA protocol implementation are presented schematically in Figure 3. Details on the reasons for rejection of eight out of 11 articles are included in Table S2.

The papers by Benech et al. (2011) [19], Arcuri et al. (2011) [23], and Kolk and Neff (2015) [20] were qualified for further analysis. The first two articles clearly indicate a common research group for the purposes of this review. Their content was synthesized. Both studies included a total of 52 applications of RA in 40 patients. Table 2 presents basic information on the nature of the studies. Due to the fact that only two groups of patients were identified and qualified, the data presented in Table 2 is only a substitute for the qualitative analysis of these records. The results of the studies by Benech et al., Arcuri et al., and Kolk and Neff are summarized in Table 3.

Despite significant differences, the studies compared by Benech and Arcuri as well as Kolk and Neff have several points of commonality. Both groups of researchers achieved a similar percentage of ideal bone fragments, reaching 90%–94% [19,20,23]. In both studies, there were no cases of persistent paralysis of the facial nerve [19,20,23]. Persistent auriculotemporal nerve dysfunctions occurred sporadically, only in the Kolk and Neff patients [19,20,23]. External acoustic meatus diameter measurements were not uniform in both groups, but it can be assumed that, despite the clinically significant strictures noted by Kolk and Neff, no strictures requiring surgical correction were found in any of the cases [19,20,23]. It should be strongly emphasized that the data resulting from the analysis of only two groups of subjects cannot be extrapolated to the entire RA method. However, they can be an important hint for future researchers and surgeons using the discussed approach.

## 4. Discussion

According to the results of the European Maxillofacial Trauma (EURMAT) project presented in 2014, mandible fractures dominate among facial bone fractures (42%). The EURMAT study by Boffano et al. was conducted on a representative group of 3396 patients from numerous centers located throughout Europe. In mandible fractures, condylar fractures constitute as much as 34%, which is the largest group identified by these authors. Boffano et al. decided to create a more detailed division of condyle fractures, distinguishing groups of extracapsular and intracapsular fractures. They cited data showing that intracapsular fractures account for about a quarter of condyle fractures, which is about 8% of all mandibular fractures [24]. Approximately 8% of mandible fractures, or over 3% of facial bone fractures, are enough to be part of the daily routine of maxillofacial surgery units. However, the given percentages apply to intracapsular condyle fractures of which the treatment is still a matter of dispute. The access to the head and the upper part of the condyle neck is protected by the skin of the aesthetic preaural area, the delicate facial nerve, and the lateral temporomandibular joint ligament. For this reason, surgical intervention within the joint has been and still is considered to be the last resort by many [7,25]. Recent years, however, have been dominated by voices supporting the need for surgical reposition and reconstruction of the height of the condyles, which is also applicable in cases of intracapsular fractures [8,26,27].

In order to surgically visualize the head or neck of the condyle, it is necessary to make a decision which tissue layers should be successively incised. Most approaches to the neck and head of the condyle begin by cutting the skin forward, up, down, or partially inside the auricle. Among the approaches to the uppermost part of the condyle, the predominant approach is the preaural one and its transaural modification. Both of them can be extended in the temporal direction. The most important, however, are the methods of preparation between the facial nerve branches. This creates the necessity to bypass or dissect and move the branches of the facial nerve. Analyzing these possibilities, the study by Imai et al. indicates that approaches passing between the branches of the facial nerve (superficial) are more secure than the surrounding (deep) ones [28]. Barham et al. specify that it is best to locate the temporozygomatic facial nerve branches division, which is a constant point of reference on the way to the condyle [29]. RA is a breakthrough in this context because it allows the complete avoidance of revealing the branches of the facial nerve, minimizing the risk of their temporary or permanent paralysis [19,20,23]. However, RA is also called the “retroauricular transmeatal approach” to emphasize the need to cut the ear canal during preparation [19,23].

The RA technique itself has been described in detail many times. The first description from 1920 comes from Bockenheimer [17]. In 1931, it was modified by Axhausen [30]. RA experienced its renaissance in 1970 thanks to Hoopes [31], whom Ivy [32] pointed out for lack of references to earlier articles. We owe the latest wave of interest in RA to Neff et al. and their 2004 and 2005 German-language articles [33,34]. Ten years later, Neff and Kolk published the most comprehensive and complete study of RA outcomes available in the English language [20]. Despite 100 years of experience in the use of RA, there is a lack of studies on patient groups. The two English-language studies found are far too few to be able to reliably assess RA. Apart from articles from 50 years ago and more, no single case reports of RA use were found.

As mentioned before, the use of RA is primarily aimed at minimizing the risk of paralysis of the facial nerve. The research of Kolk and Neff as well as Benech and Arcuri are consistent in this matter. In none of the 40 described patients, persistent paralysis of any branch of the facial nerve was found. In both groups, temporary paralysis of this nerve occurred sporadically. Knowing the nature of facial nerve palsy, it is easy to understand that cases of palsy lasting up to six months can be attributed to neuropraxia (a type of contusion) or axonotmesis (rupture of the internal parts of the nerve, usually due to stretching). This means that they were likely caused by pressure and stretching of the delicate nerve branches, indicating the indirect nature of the iatrogenic injury. The course of RA itself, if the surgical technique is correct, practically excludes the possibility of severing the facial nerve branches, which is one of the greatest advantages of this approach. In turn, temporary or permanent paralysis of the sensory auriculotemporal nerve appears to be a typical complication of RA. Data on this subject are divergent and indicate 0%–10% of persistent paralysis [19,20,23], but the observations of Kolk and Neff indicate as much as 42% of temporary paralysis [20]. This proves the real risk of loss of sensation in the anterior segment of the auricle and the temporal area. Therefore, the possibility of occurrence of this complication should be carefully discussed with the patient when considering possible therapeutic strategies.

Moreover, RA requires cutting the auricular canal, which must be done very carefully to be able to reconstruct the canal correctly after the fixation is finished. Cutting and then suturing the ear canal implies the possibility of having canal stenosis. To prevent this from happening, a special surgical suturing protocol is used, precisely described and illustrated by Neff [18], and an earplug is used in the postoperative period. Despite these preventive measures, according to Kolk and Neff, a slight (up to 1 mm loss in diameter) or moderate (up to 1.5 mm loss in diameter) stenosis occurs in about one in three cases [20]. These authors did not report any more severe auricular canal stenosis [20]. Similar observations were presented by Benech and Arcuri. In their material, no patient was found with a major reduction in the diameter of the auricular canal [19,23].

Furthermore, RA saves the ligaments of the lateral pole of the mandibular head. Leaving these structures intact is impossible with other approaches, where, in order to visualize the head, it is then necessary to cut and delaminate the lateral ligament, damage the lateral part of the joint capsule, and, to some extent, even impair disc ligaments. The destructive force of this stage of surgery for the temporomandibular joint seems to be high, but its consequences are still unclear due to the constantly developed views on the role of the previously mentioned ligaments [35]. Part of the explanation comes from observation of Kolk and Neff who note no deterioration in protrusive mandibular movement in the group of their patients treated with RA, which the authors justify by not injuring the ligaments surrounding the lateral pole of the mandibular head [20]. Moreover, it is known that leaving material for osteosynthesis protruding from the mandibular head surface leads to chronic irritation of the joint structures [27]. Contact of such irritants with the articular disc attachments and the articular capsule may lead to scarring of these structures, which impairs the mobility of the temporomandibular joint [27]. This explains the superiority of open treatment over closed treatment in the context of maintaining the mobility of the mandible, as well as the superiority of RA over preaural approaches [8,9,10]. Moreover, it is worth emphasizing about 30% higher values of mandibular abduction in the Kolk and Neff study compared to the results achieved by Benech et al. and Arcuri et al. [19,20,23]. This may be due to the use of mainly long screws embedded in the bone by the first authors compared to the use of miniplates in the group studied by the second group of researchers [19,20,23].

Complete separation of the mandibular head leads to rupture of vessels from the base of the condyle, i.e., the temporomandibular artery. The temporomandibular artery branches out from the inferior alveolar artery. The temporomandibular artery runs intraosseously parallel to the inferior alveolar artery in a short distance, and then turns inferiorly toward mandibular base and immediately superiorly straight into the mandibular head. At the junction of the cervix to the mandibular head, the temporomandibular artery divides into a series of smaller branches, which vascularize the mandibular head [36,37,38]. In such situations, head necrosis is prevented by collateral circulation, largely from the superficial temporal artery. However, the scope and origin of the collateral circulation are characterized by high anatomical variability [36,38]. The course of the superficial temporal artery enables relatively safe operation of low fractures, but is unfavorable for all approaches to the head of the mandible. This is noteworthy in the context of RA, during which this vessel may be injured. Apart from the risk of necrosis of the mandibular head, such an injury may lead to massive bleeding, and, in extreme cases, prolonged bleeding [37].

Despite the posterolateral view, RA provides very good conditions for fixation of the mandibular head due to the typical course of the fracture line [38,39]. However, the course of typical neck fracture lines are less favorable for the use of RA [39]. The non-articular surface of the head, well visualized thanks to RA, can be used to attach microplates or miniplates, which some authors recommend to remove after trans-head placement of the final screws [19,20,23].

The most difficult thing to refer to is the choice of fixation material and the consequences of this choice. All sources known to us agree that the articular surface of the mandibular head should be free from unevenness caused by the insertion of screws or miniplates and microplates in the mandibular head. The possibility of fusing the mandibular head without miniplates or microplates is emphasized by, among others, Neff and Kolk as well as Kozakiewicz [20,40]. Moreover, according to Kozakiewicz, the value of headless screws, which can be completely embedded in the bone, should be emphasized [41]. Kolk and Neff’s attention to positioning the screw heads in such a way that they do not interfere with the work of the joint seems to be of clear benefit [20,34]. Compared to the Benech et al. and Arcuri et al. study, Kolk and Neff achieved approximately 32% greater long-term mouth opening, i.e., 50 mm versus 38 mm [19,20,23]. This may be due to the fact that Benech et al. and Arcuri et al. used mini-plates each time, whereas Neff and Kolk only occasionally left microplates in addition to large screws with heads positioned favorably for joint function [19,20,23].

There are many long screw systems dedicated to head fractures [33,34,39,40,41]. Due to the anatomy of the head and the direction of insertion of the screws from the RA, screws with a length of 14 to 18 mm are usually used [33,34,39,40,41]. Screws with diameters from 1.5 to 2.5 mm dominate of which the thicker ones are often cannulated and inserted along the Kirschner wire [33,39,40,41]. The construction of the screws, especially the shape of their heads and the arrangement of the thread lamellas, result in different tightening and compression forces of bone fragments [33,34,39,40,41]. Research is being conducted on the types and number of screws necessary for a sufficient fracture stabilization, which is due to the non-cortical nature of the mandibular head. This is unique in the scale of the entire skeleton and quite difficult to simulate in vitro [33,40,41]. It is assumed that, due to anatomical limitations, up to three screws are inserted, and each subsequent one is an unquestionable benefit for the quality of the fixation [33,34,39,40,41]. According to Kolk and Neff, in the case of an unfavorable fracture fissure and the presence of intermediate fragments, additional use of microplates are arranged so as to minimize interference with the joint structures [20,34]. Nevertheless, it is most advantageous to embed all the fixative material in the structure of the mandibular head [20,39,40,41,42,43]. To achieve this, headless screws were introduced, which are assumed to be less irritating to the lateral ligament and, thus, reduce the risk of resorption of the mandibular head [39,40,42,43].

Therefore, there is a motivated temptation to leave headless screws as they may not generate functional problems in the future. In this context, one should recall the concern of Kolk and Neff that the leftover material for osteosynthesis undergoes osseointegration with time and may be much more difficult to remove [20]. According to Kolk and Neff, the most appropriate time for removal of osteosynthesis material is not long after the completion of bone regeneration, i.e., about 6 months after the surgery [20]. Consequently, the next step to improve the fixation techniques is the gradual introduction of resorbable screws, including those made of magenz alloys and self-reinforcing polymers, which could be the future of a mandibular head fracture surgery [39].

## 5. Conclusions

RA may be the method of choice due to the bypass of the facial nerve in cases where surgical treatment of mandibular condyle head fractures is required. Nevertheless, we must always remember the consequences of applying each open approach, which is the extreme opposite in relation to conservative treatment. On one hand, open treatment almost always perfectly restores the correct position of bone fragments, but, on the other hand, it implies the intersection of structures in the course of the approach with all its consequences. Therefore, when choosing RA, the functional consequences and reduced quality of life associated with possible auriculotemporal nerve paralysis and auricular canal stenosis should be carefully discussed with the patient. Respectively, the fixation technique itself, with the choice of fixation material at the forefront, is currently the subject of a lively discussion among authorities and is clearly evolving, producing solutions that have a smaller effect on the function of the temporomandibular joint.

## Figures and Tables

**Table 1:** Inclusion and exclusion criteria. Description in the text.

	Inclusion Criteria	Exclusion Criteria
Population	Patients with mandibular condylar fracture	Animal patients
Intervention	Surgical management using retroauricular approach	-
Comparison	Any or none	-
Outcomes	Reduction with or without fixation	-
Study design	Original papers	non-English papers

**Table 2:** Information on the type of studies analyzed.

Authors, Date of Publication	Method of Analysis	Number of Condyles (Patients) Operated Via RA	Presence of a Control Group
Benech et al. 2011 [19] Arcuri et al. 2011 [23]	retrospective	16 (14)	No
Kolk and Neff 2015 [20]	prospective	36 (26)	Yes

**Table 3:** Results of the analyzed studies.

Authors	Benech et al., Arcuri et al. [19,23]	Kolk and Neff [20]
Duration of operation, average	29–67 min, avg. 43 min	unknown
Fixing material used	screwed miniplates	screws with no miniplates and in some cases also screwed microplates
Mean maximal interincisal mouth opening after a minimum of one year	38 mm	50 mm
Percentage of condyles with long-term ideal bone fragment position	94%	90%
Percentage of patients with long-term no or minimal pain	unknown	90%
Percentage of patients with no noticeable long-term defects in facial nerve function	100%	100%
Percentage of patients with no noticeable long-term defects in auriculotemporal nerve function	100%	90%
Percentage of patients with no clinically relevant auricular stenosis	100%	89%

## Supplementary Materials

The following are available online at https://www.mdpi.com/2077-0383/10/2/230/s1, Table S1: Details of the search strategies using PubMed, BASE and Google Scholar search engines, Table S2: Records screened.
