# Supplementary material for: Retro-Auricular Approach to the Fractures of the Mandibular Condyle: A Systematic Review
Source: J Clin Med. 2021 Jan 11;10(2):230. doi: 10.3390/jcm10020230 (PMC7826936; doi:10.3390/jcm10020230)
Supplement: Supplementary file 1 [file jcm-10-00230-s001.pdf]

Table S1. Details of the search strategies using PubMed, BASE and Google Scholar search engines.

	Search strategy
PubMed	(mandible OR mandibular) AND (head OR neck OR collum OR condyle OR condylar) AND (fracture OR fractures) AND (retroauricular OR retro-auricular OR retroaural OR retro-aural OR postauricular OR post-auricular OR postaural OR post-aural OR transmeatal OR trans-meatal) AND (approach OR access)
BASE	(mandible mandibular) AND (head neck collum condyle condylar) AND (fracture fractures) AND (retroauricular retro-auricular retroaural retro-aural postauricular post-auricular postaural post-aural transmeatal trans-meatal) AND (approach access)
Google Scholar	allintitle: (mandible OR mandibular) (head OR neck OR collum OR condyle OR condylar) (retroauricular OR retro-auricular OR retroaural OR retro-aural OR postauricular OR post-auricular OR postaural OR post-aural OR transmeatal OR trans-meatal)

Table S2. Records screened.

Record (authors, title, date of publication)	Reason for exclusion (if applicable)
Kolk A, Neff A. Long-term results of ORIF of condylar head fractures of the mandible: A prospective 5-year follow-up study of small-fragment positional-screw osteosynthesis (SFPSO). 2015	Included
Hoopes JE, Wolfort FG, Jabaley ME. Operative treatment of fractures of the mandibular condyle in children. Using the post-auricular approach. 1970	Wrong publication date
Ivy RH. Post-auricular approach to mandibular condyle. 1970	Wrong publication date
Arcuri F, Brucoli M, Benech A. Analysis of the retroauricular transmeatal approach: a novel transfacial access to the mandibular skeleton. 2012	Included
Baek RM, Min KH, Heo CY, Eun SC. The perilobule approach to subcondylar fractures. 2011	Wrong intervention
Neff A, Mühlberger G, Karoglan M, et al. Stabilität der Osteosynthese bei Gelenkwalzenfrakturen in Klinik und biomechanischer Simulation. 2004	Wrong language
Benech A, Arcuri F, Baragiotta N, Nicolotti M, Brucoli M. Retroauricular transmeatal approach to manage mandibular condylar head fractures. 2011	Included
García-de Marcos JA. Minimal Incision Approach for Condyle Fracture Treatment. 2018	Wrong intervention
Al-Moraissi EA, Louvrier A, Colletti G, et al. Does the surgical approach for treating mandibular condylar fractures affect the rate of seventh cranial nerve injuries? A systematic review and meta-analysis based on a new classification for surgical approaches. 2018	Wrong type of article

Duckwitz D. Unfall oder Gewaltdelikt? Retrospektive Analyse von Gesichtsverletzungen verursacht durch stumpfe Gewalt. 2014	Wrong language
Kim WS, Jittreetat T, Nam W, Sannikorn P, Choi EC, Koh YW. Reconstruction of the segmental mandibular defect using a retroauricular or modified face-lift incision with an intraoral approach in head and neck cancer. 2015	Wrong population
